# From Challenge to Opportunity: ABO-Incompatible Kidney Transplantation to Expand the Donor Pool at a Single Center

**DOI:** 10.7759/cureus.113195

**Published:** 2026-07-22

**Authors:** Dinesh Khullar, Neelam Devi Maravi, Deepak Kumar Panigrahi, Abhishek, Kulwant Singh, Simran Sawhney, Sahil Bagai, Ankur Laskar, Anthony J Gyunda, Abhijit Chavan, Mehvish Khan, Nikita Matolia, Rahul Grover, Manu Prakash, Narinder Pal Singh, Anish Kumar Gupta

**Affiliations:** 1 Department of Nephrology and Renal Transplant Medicine, Max Super Speciality Hospital, New Delhi, IND

**Keywords:** abo-incompatible transplantation, desensitization protocols, donor pool expansion, graft outcomes, kidney transplantation

## Abstract

Introduction

ABO-incompatible kidney transplantation (ABOiKT) has emerged as a viable option whenever there is non-availability of a compatible donor. Traditionally considered a contraindication due to the high risk of antibody-mediated rejection, advancements in desensitization protocols, immunosuppressive therapy, and post-transplant monitoring have significantly improved the outcomes. In this study, we have detailed our experience of ABOiKT performed in a tertiary care hospital in North India.

Methodology

It was a single-center retrospective cohort study. All consecutive ABOiKT performed from 2014 to 2021 were screened. Recipients younger than 15 years of age, with dual-organ transplants, and with highly sensitized transplants were excluded from the study. Three years of follow-up data from the hospital records were collected and analyzed. The primary objective was to study the incidence of biopsy-proven acute rejections (BPARs), and secondary objectives were to study the incidence of graft loss and infectious complications.

Results

A total of 93 ABOiKTs meeting the inclusion and exclusion criteria were included in the study. Fourteen (15.1%) kidney transplant recipients (KTRs) experienced BPAR, the majority of which were antibody-mediated (10 out of 14; 71.42%). Overall, 8 (8.6%) KTRs experienced graft loss. The rejection-free survival at six months, one year, and three years was 84 (90.3%), 83 (89.2%), and 79 (84.9%), respectively. Correspondingly, graft survival at six months, one year, and three years was 90 (96.8%), 87 (93.5%), and 85 (91.4%), respectively. Urinary tract infection was the most common infection encountered in 35 (37.6%) cases. There was no significant difference in incidence of acute rejection or graft loss between the two cohorts with basiliximab/nil or rabbit antithymocyte globulin as the induction agent. In regression analysis, anti-A/B antibody titer or the type of induction agent used did not predict acute rejection; however, delayed graft function was significantly associated with rejection episodes (OR: 43.33, 95% CI: 4.54-413.27, p<0.05).

Conclusions

With careful recipient selection and vigilant post-transplant monitoring, patient and graft outcomes can be improved substantially after ABOiKT. This approach offers a promising solution to reduce waiting times and expand the donor pool for potential KTRs.

## Introduction

Kidney transplantation (KT) is the best form of renal replacement therapy available to a suitable candidate [1]. Transplantation strategies have evolved over time. Since long, blood group incompatibility has been considered a major barrier to KT. The initial reluctance to pursue ABO-incompatible KT (ABOiKT) stemmed from the high immunological risk associated with pre-existing anti-A/B antibodies, leading to concerns about hyperacute rejection and poor graft outcomes. But with the evolution of the art of KT, ABOiKT has emerged as a viable option for patients with end-stage kidney disease, expanding the donor pool and offering hope to the waitlisted patients. As a result, ABOiKT is now routinely performed in many centers worldwide, with outcomes in select cohorts approaching those of ABO-compatible KT (ABOcKT) [2,3]. Despite advancements in immunosuppressive therapies and desensitization protocols, ABOiKT poses challenges, including increased risk of rejection and graft failure, especially in the initial period [4]. And not to forget the risk of infectious complications after ABOiKT, whose incidence has been found to be higher than that of ABOcKT [5]. In India, ABOiKT has been routinely performed for the past 12 years. However, there are unique challenges such as cost constraints, limited access to advanced immunological monitoring, variability in desensitization protocols, and an increased burden of infectious complications due to environmental and socioeconomic factors. In India, we have a scarcity of robust follow-up data as far as ABOiKT is concerned. Although multicenter and single-center experiences have demonstrated encouraging outcomes, there remains a need for additional long-term data from Indian centers to better characterize patient and graft survival, rejection patterns, infectious complications, and protocol effectiveness in real-world practice. In this study, we have analyzed the follow-up data from ABOiKT performed at a tertiary care center in North India. Though it is retrospective, the relatively large cohort and standardized institutional protocol would provide valuable insights into the feasibility, safety, and long-term effectiveness of ABOiKT in the Indian setting, thereby helping to strengthen the evidence base and guide future transplant practices in the country.

## Materials and methods

This was a single-center, retrospective, cohort study. This study was conducted and reported in accordance with the Strengthening the Reporting of Observational Studies in Epidemiology (STROBE) guidelines. Kidney transplant recipients (KTRs) who underwent ABOiKT at the study center between 2014 and 2021 were screened. Eligible participants were required to have documented pre-transplant immunological evaluation, including ABO antibody titers and crossmatch testing, and a minimum of three years of follow-up, with availability of complete clinical records, including demographic, biochemical, immunological, and transplant-related data. Only first or repeat kidney-only ABOiKT performed using standard institutional desensitization and immunosuppression protocols were considered. Patients aged 15 years or older at the time of transplantation were included. Recipients who underwent dual-organ or multiorgan transplantation and highly sensitized KTR with a positive complement-dependent cytotoxicity or flow cytometry crossmatch were excluded. Patients with ABOcKT and those with incomplete medical records or inadequate follow-up data were also excluded from the analysis. A total of 110 ABOiKT recipients were screened for eligibility. Of these, 93 patients fulfilled the predefined inclusion and exclusion criteria and were included in the final analysis.

The primary objective was to study the incidence of biopsy-proven acute rejection (BPAR) episodes, and the secondary objectives were to study the incidence of graft loss and various infectious complications. All necessary clinical and biochemical data were collected retrospectively from the hospital records.

**Figure 1:**
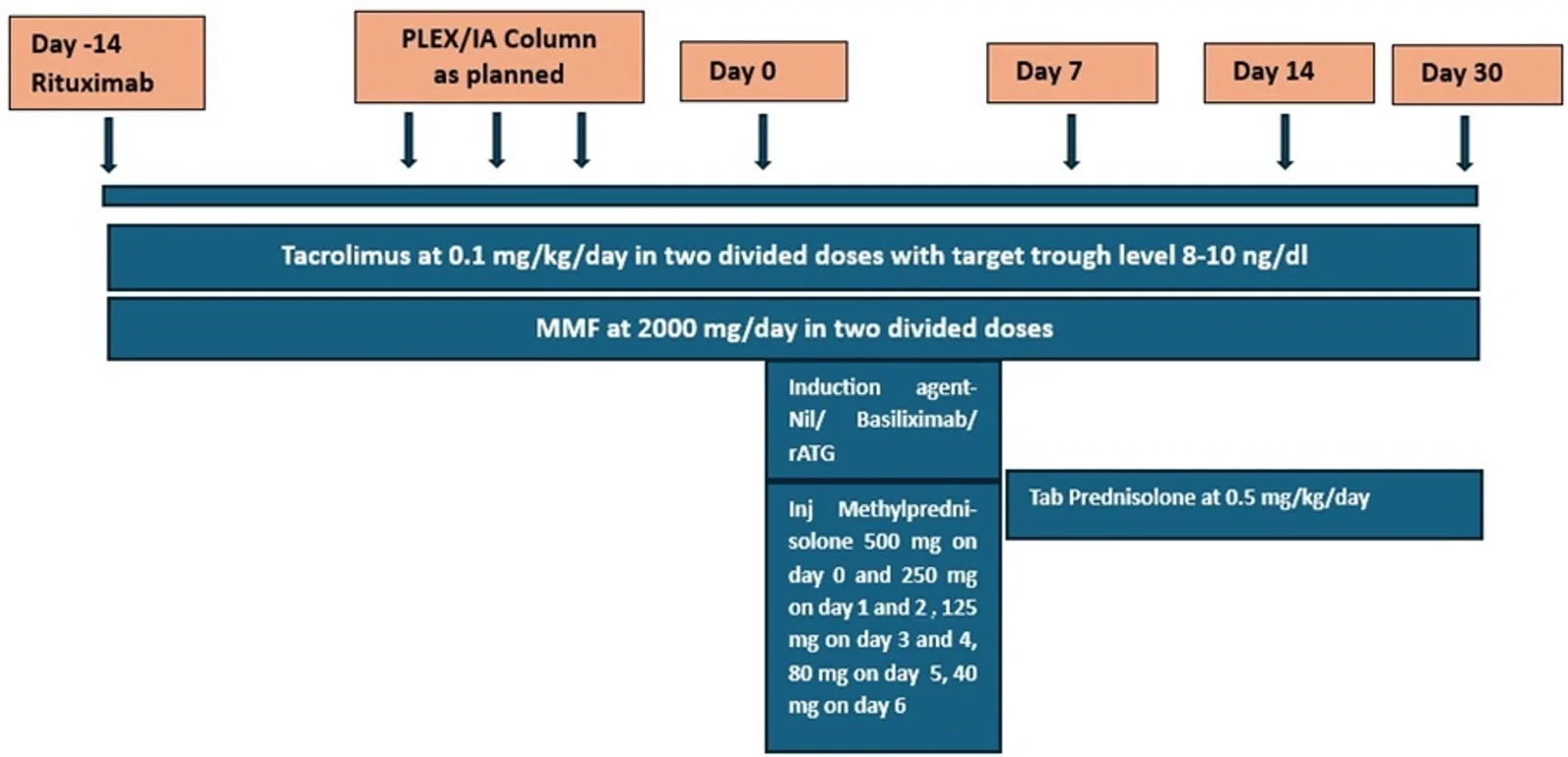
Schema of ABOiKT protocol ABOiKT, ABO-incompatible kidney transplantation; IA, immunoadsorption; MMF, mycophenolate mofetil; PLEX, plasma exchange; rATG, rabbit antithymocyte globulin.

All KTRs received a single dose of rituximab injection two weeks before transplantation. In the initial phase, we had used rituximab at a dose of 500 mg, and subsequently, from 2015 onwards, we have been using it at a dose of 200 mg in accordance with the change in international standards. On the following day, patients were commenced on tacrolimus at 0.1 mg/kg/day in two divided doses, aiming for trough levels of 8-10 ng/dL. Additionally, all recipients were prescribed either mycophenolate mofetil (MMF) (1,000 mg twice daily) or enteric-coated mycophenolic acid (720 mg twice daily) after rituximab administration (Figure 1).

We used an immunoadsorption (IA) column if the anti-A/B titer was more than 1:32. We did plasmapheresis with donor blood group plasma replacement if the titer was 1:32 or less. The target anti-A/B titer before transplant was 1:8 or less. In some cases, both IA columns and plasmapheresis were used. Anti-A/B antibody titer was monitored twice daily till post-operative day 5. Rabbit antithymocyte globulin (rATG) induction was used in the majority of cases till the year 2019; however, since 2020, we have been using injection basiliximab. In some selected cases, we did not use any induction agents. Injectable methyl prednisolone 500 mg was given to all KTRs on the day of surgery, followed by gradual tapering of injection methyl prednisolone doses, and by post-operative day 5, oral prednisolone was started at 0.5 mg/kg/day. This dose was tapered to 15-20 mg in one month and then gradually to 5 mg per day by the end of the third month post-transplant. All patients received triple-drug immunosuppression, including prednisolone, a calcineurin inhibitor (tacrolimus or cyclosporine), and mycophenolic acid formulations (MMF or enteric-coated mycophenolate sodium). Post-transplant follow-up was scheduled twice weekly during the first month, weekly for the next three months, monthly for the following six months, and every two months thereafter. Anti-A/B antibody titer was assessed twice weekly only during the first two weeks after discharge. Universal prophylaxis included oral valganciclovir for three months and cotrimoxazole for 12 months. Infectious complications were managed with antibiotics guided by culture results and local resistance patterns. Delayed graft function (DGF) was defined as the requirement for hemodialysis within the first post-transplant week. Protocol biopsies were not performed. Rejection was diagnosed as per the latest available Banff criteria. Acute cellular rejection (ACR) episodes were treated with methylprednisolone pulses, with rATG being administered when required, whereas antibody-mediated rejection (AMR) was managed using plasmapheresis and intravenous immunoglobulin. Graft loss was defined as the need for resumption of permanent renal replacement therapy or graft nephrectomy or death with functioning graft or a sustained fall in estimated glomerular filtration rate below 15 mL/min/1.73 m².

Ethical considerations

Ethical approval for the study was obtained from the Ethics Committee of Max Healthcare, New Delhi (approval number: BHR/RS/MSSH/MHIL/SKT-1/MHEC/NEPHRO/25-07). The research was conducted in accordance with the principles of the Declaration of Helsinki and in compliance with Good Clinical Practice guidelines.

Statistical analysis

Data analysis was performed using SPSS software, version 22.0 (IBM Corp., Armonk, NY, USA). Categorical variables were expressed as frequencies and percentages, while continuous variables were summarized as mean ± standard deviation or median with interquartile range. Comparisons between categorical variables were conducted using the chi-square test or Fisher’s exact test when appropriate. For continuous data, the independent t-test was applied to normally distributed variables, and the Wilcoxon rank-sum test was used for non-normally distributed variables. Logistic regression was employed to identify predictors of acute rejection. A p-value <0.05 was considered statistically significant.

## Results

We analyzed the data of 93 ABOiKTRs, and the baseline characteristics of recipients and donors are presented in Table 1. The mean ages of the recipients and donors were 40.90 ± 11.94 and 46.70 ± 10.00 years, respectively. Seventy-nine (84.9%) recipients were males, and 73 (78.5%) donors were females. The median anti-A/B antibody titer was 32 (IQR: 8.64). In the initial period of the study, rituximab was used at a dose of 500 mg. Subsequently, it was administered at a dose of 200 mg.

**Table 1:** Demographic characteristics of the recipients and donors (N=93) ADPKD, autosomal dominant polycystic kidney disease; BMI, body mass index; CGN, chronic glomerulonephritis; CTID, chronic tubulointerstitial disease; DN, diabetic nephropathy; GFR, glomerular filtration rate; HLA, human leucocyte antigen; IQR, interquartile range; rATG, rabbit antithymocyte globulin; SD, standard deviation.

Parameter	N=93
Recipient age in years, mean (SD)	40.90 (11.94)
Recipient gender, N (%)	
Males	79 (84.9)
Females	14 (15.1)
BMI in kg/m^2^, mean (SD)	23.65 (3.49)
Basic kidney disease, N (%)	
CGN	16 (17.2)
CTID	6 (6.5)
DN	22 (23.7)
ADPKD	4 (4.3)
Unclassified	45 (48.4)
Associated comorbidities, N (%)	
Diabetes mellitus	23 (24.7)
Hypertension	69 (74.2)
Hypothyroidism	11 (11.8)
Hepatitis B infection	02 (2.2)
Hepatitis C infection	04 (4.4)
Re-transplantation, N (%)	6 (6.5)
Dialysis vintage in years, median (IQR)	3 (1-10)
Recipient blood group, N (%)	
A	20 (21.5)
B	22 (23.7)
O	51 (54.8)
Donor age in years, mean (SD)	46.70 (10)
Donor gender, N (%)	
Males	20 (21.5)
Females	73 (78.5)
Donor blood group, N (%)	
A	30 (32.3)
B	48 (51.6)
AB	15 (16.1)
Anti-A/B titer median (IQR)	32 (8-64)
Donor GFR, mean (SD)	44.75 (6.38)
Biological relationship, N (%) unrelated	47 (50.53)
Number of HLA mismatch, mean (SD)	3.54 ± 1.42
Rituximab, N (%)	
200 mg	87 (93.5)
500 mg	6 (6.5)
Antibody removal, N (%)	
Immunoadsorption	23 (24.73)
Plasmapheresis	39 (41.93)
Both	17 (18.27)
None	14 (15.05)
Induction agent, N (%)	
Nil	7 (7.5)
Basiliximab	12 (12.9)
rATG	74 (79.6)

The clinical outcome data are presented in Table 2. DGF was encountered in 6 (6.5%) recipients of the cohort. Fourteen KTRs (15.1%) had BPAR. The majority of them (10 out of 14; 71.42%) were AMR. Three (21.4 %) were mixed, and one (7.1%) was ACR. A total of 8 (8.6%) KTRs had graft loss during the three-year follow-up. At the end of follow-up, two (2.14%) recipients had died with functioning grafts, one due to a cardiac event and the other due to infective complications. The death-censored graft loss rate was 6.45% (6/93). Among the six graft losses, two were secondary to BK virus nephropathy and four to chronic rejection.

**Table 2:** Clinical outcomes of kidney transplant recipients (n=93) BKV, BK virus; CMV, cytomegalovirus; GN, glomerulonephritis; LRTI, lower respiratory tract infection; PTDM, post-transplant diabetes mellitus; SD, standard deviation; UTI, urinary tract infection.

Clinical outcome	Value
Serum creatinine (mg/dL), mean (SD)	
At discharge	1.16 (0.48)
At 1 month	1.32 (0.72)
At 6 months	1.44 (0.73)
At 1 year	1.50 (1.01)
At 3 years	1.47 (0.62)
Delayed graft function, N (%)	6 (6.5)
PTDM, N (%)	33 (35.5)
Bacterial UTI, N (%)	35 (37.6)
Bacterial LRTI, N (%)	11 (11.8)
Tuberculosis, N (%)	3 (3.2)
Other bacterial infections, N (%)	20 (21.5)
Fungal infections, N (%)	3 (3.2)
CMV disease, N (%)	7 (7.5)
BKV nephropathy, N (%)	7 (7.5)
Parvo virus infections, N (%)	3 (3.2)
Malignancy, N (%)	1 (1.07)
Recurrent GN, N (%)	4 (4.3)
Leukocytopenia	18 (19.4)
Biopsy-proven acute rejection, N (%)	14 (15.1)
Graft loss, N (%)	8 (8.6)
Death, N (%)	2 (2.14)
Death-censored graft loss, N (%)	6 (6.45)

All specific outcomes, including BPAR, death, graft loss, death-censored graft loss, and infective episodes with respect to the time frame, are shown in Tables 3, 4. The rejection-free survival at six months, one year, and three years was 84 (90.3%), 83 (89.2%), and 79 (84.9%), respectively. Similarly, the overall graft survival at six months, one year, and three years was 90 (96.8%), 87 (93.5%), and 85 (91.45%), respectively. Bacterial infections, especially urinary tract infections (UTIs), were the most common infective complications encountered in the post-transplant period.

**Table 3:** Post-transplant outcomes with respect to time period (n=93)

Endpoint	Total, n (%)
Acute rejection episodes, n (%)	14 (15.1)
Immediate post-operative period	9 (9.7)
From discharge up to the end of 30 days	0 (0)
From 2nd month up to the end of 6th month	0 (0)
From 7th month up to the end of 1 year	1 (1.1)
From 2nd year up to the end of 3rd year	4 (4.3)
Total graft loss, n (%)	8 (8.6)
Immediate post-operative period	1 (1.1)
From discharge up to the end of 30 days	1 (1.1)
From 2nd month up to the end of 6th month	1 (1.1)
From 7th month up to the end of 1 year	3 (3.2)
From 2nd year up to the end of 3rd year	2 (2.2)
Death, n (%)	2 (2.1)
Immediate post-operative period	0 (0)
From discharge up to the end of 30 days	0 (0)
From 2nd month up to the end of 6th month	0 (0)
From 7th month up to the end of 1 year	0 (0)
From 2nd year up to the end of 3rd year	2 (2.1)
Death-censored graft loss, n (%)	6 (6.4)
Immediate post-operative period	1 (1.0)
From discharge up to the end of 30 days	1 (1.0)
From 2nd month up to the end of 6th month	1 (1.0)
From 7th month up to the end of 1 year	3 (3.4)
From 2nd year up to the end of 3rd year	0 (0)

**Table 4:** Infectious complications with respect to time period (n=93) BKV, BK virus; CMV, cytomegalovirus.

Timepoint	N (%)
Immediate postoperative period	
Total bacterial infection	22 (23.65)
Fungal infection	1 (1.07)
CMV infection	0 (0)
BKV infection	0 (0)
From discharge up to the end of 30 days	
Total bacterial infection	8 (8.60)
Fungal infection	1 (1.07)
CMV infection	0 (0)
BKV infection	0 (0)
From 2nd month up to the end of 6th month	
Total bacterial infection	15 (16.12)
Fungal infection	1 (1.07)
CMV infection	3 (3.22)
BKV infection	5 (5.37)
From 7th month up to the end of 1 year	
Total bacterial infection	11 (11.82)
Fungal infection	0 (0)
CMV infection	4 (4.30)
BKV infection	2 (2.15)
From 2nd year up to the end of 3rd year	
Total bacterial infection	10 (10.75)
Fungal infection	0 (0)
CMV infection	1 (1.07)
BKV infection	1 (1.07)

When we compared the data of KTR in whom rATG was used as the induction agent against those with either basiliximab or no induction, we could not find significant differences with respect to the general characteristics, BPAR, graft loss, or other infectious or non-infectious complications except for the significantly increased incidence of leukopenia in the rATG group (Table 5).

**Table 5:** Comparison of baseline characteristics and clinical outcomes among kidney transplant recipients according to induction therapy (no induction/basiliximab vs. rATG) BMI, body mass index; BKV, BK virus; BPAR, biopsy-proven acute rejection; CMV, cytomegalovirus; DGF, delayed graft function; HLA, human leukocyte antigen; IQR, interquartile range; KTR, kidney transplant recipient; LRTI, lower respiratory tract infection; rATG, rabbit antithymocyte globulin; SD, standard deviation; UTI, urinary tract infection.

Parameter	Nil/basiliximab (N=19)	rATG (N=74)	p-value
Recipient age (mean ± SD)	38.53 ± 11.57	41.51 ± 12.04	0.334
Recipient gender, N (%)			
Males	17 (89.5)	62 (83.8)	0.536
Females	02 (10.5)	12 (16.2)	
BMI (mean ± SD)	24.11 ± 3.47	23.52 ± 3.51	0.524
Dialysis vintage, median (IQR)	6 (2-12)	3 (1-9.25)	0.168
Anti-A/B titer, median (IQR)	16 (8-128)	32 (8-64)	0.573
HLA mismatch (mean ± SD)	3.32 ± 1.45	3.59 ± 1.42	0.450
Plasmapheresis, N (%)	14 (73.7)	42 (56.8)	0.179
Immunoadsorption, N (%)	7 (36.8)	33 (44.6)	0.543
NODAT, N (%)	5 (26.3)	28 (37.8)	0.349
Bacterial UTI, N (%)	9 (47.4)	26 (35.1)	0.326
Bacterial LRTI, N (%)	1 (5.3)	10 (13.5)	0.321
Other bacterial infections, N (%)	3 (15.8)	17 (23)	0.497
Fungal infections, N (%)	0	3 (4.1)	0.372
CMV disease, N (%)	1 (5.3)	6 (5.1)	0.675
BKV nephropathy, N (%)	2 (10.5)	5 (6.8)	0.579
Parvovirus infection, N (%)	0	3 (4.1)	0.372
Leukopenia, N (%)	0	18 (24.3)	0.017
DGF, N (%)	2 (10.5)	4 (5.4)	0.418
BPAR, N (%)	2 (10.5)	12 (16.2)	0.536
Graft loss, N (%)	1 (5.3)	7 (9.5)	0.561

**Figure 2:**
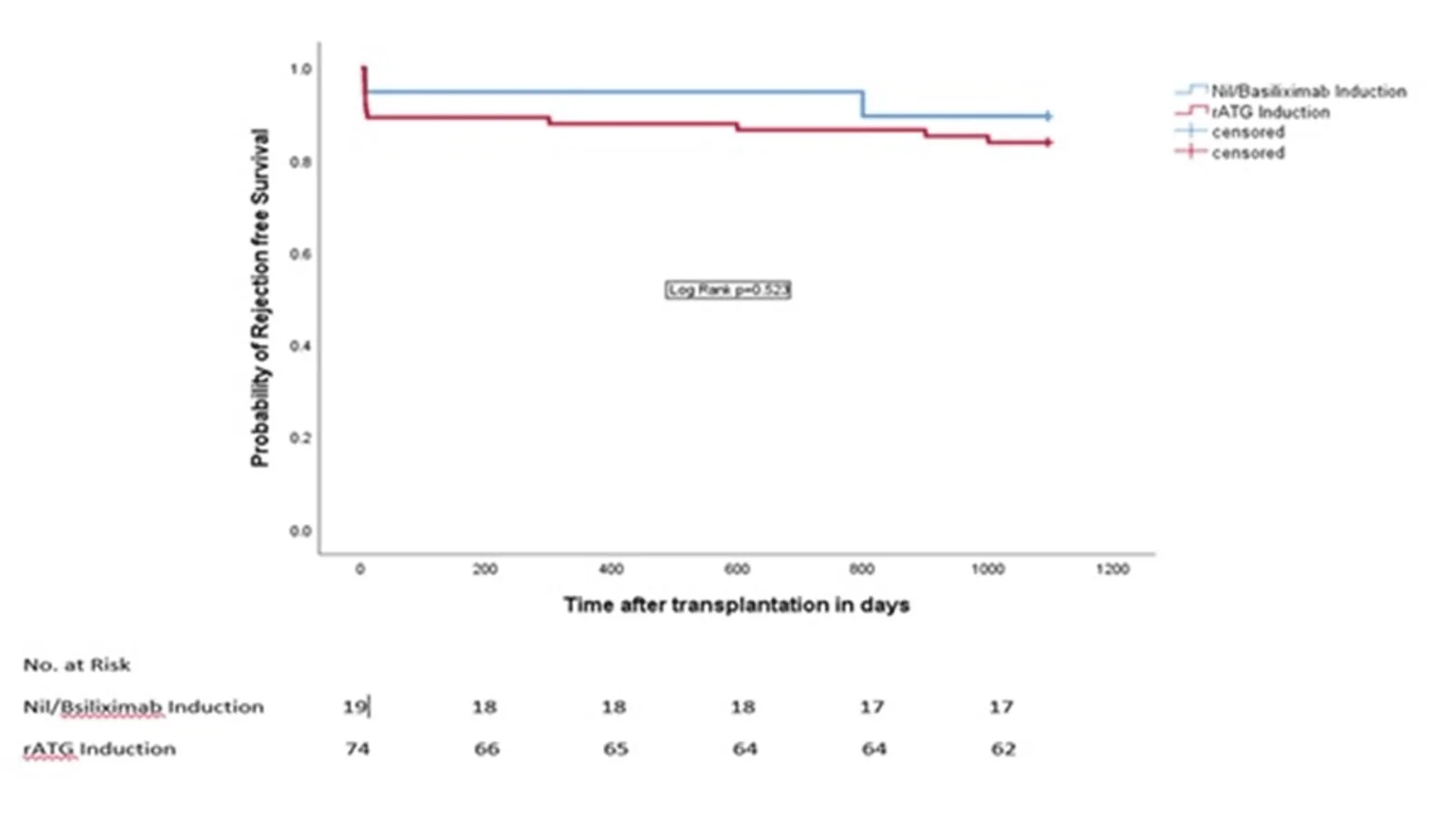
Kaplan-Meier survival estimates for rejection-free survival with the type of induction agents used rATG, rabbit antithymocyte globulin.

**Figure 3:**
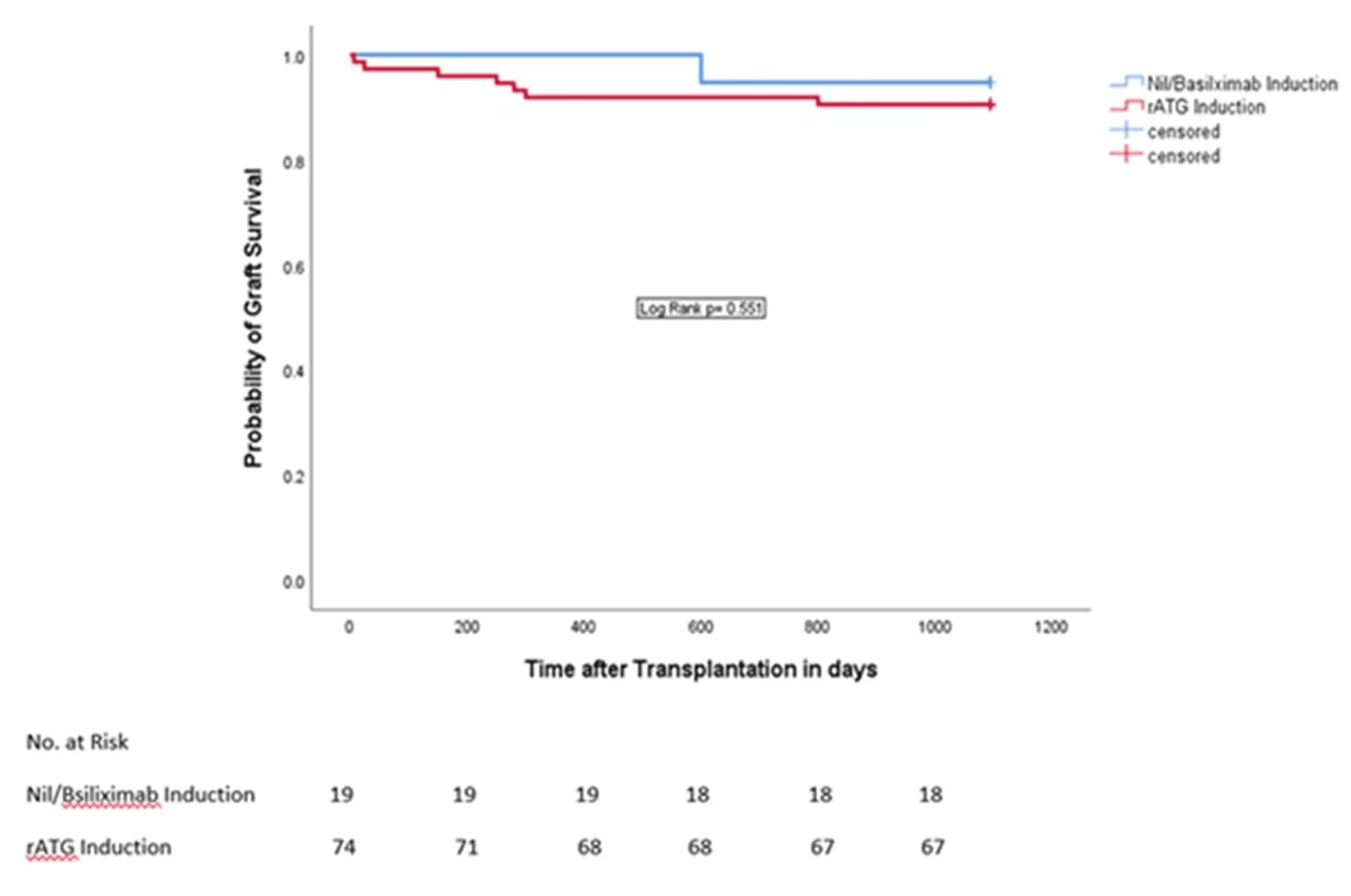
Kaplan-Meier survival estimates for graft survival with the type of induction agents used rATG, rabbit antithymocyte globulin.

On Kaplan-Meier survival analysis, there was no difference in rejection-free survival or graft survival between the two groups (Figures 2, 3).

The incidence of DGF and graft loss was significantly higher in those with BPAR (Table 6). The incidence of DGF was also significantly higher among those with ABOiKT whose baseline anti-A/B titer was >1:32 (Table 7).

**Table 6:** Comparison of general characteristics and outcomes between KTR with and without rejections BKV, BK virus; BMI, body mass index; BPAR, biopsy-proven acute rejection; CMV, cytomegalovirus; DGF, delayed graft function; HLA, human leukocyte antigen; IA, immunoadsorption; IQR, interquartile range; LRTI, lower respiratory tract infection; PTDM, post-transplant diabetes mellitus; rATG, rabbit antithymocyte globulin; SD, standard deviation; UTI, urinary tract infection.

Parameter	No BPAR (N=79)	BPAR (N=14)	p-value
Recipient age (mean ± SD)	40.87 ± 11.98	41.07 ± 12.14	0.955
Recipient gender, N (%)			
Males	68 (86.1)	11 (78.6)	0.469
Females	11 (13.9)	03 (21.4)	
BMI (mean ± SD)	23.53 ± 3.44	24.30 ± 3.84	0.452
Dialysis vintage, median (IQR)	3 (1-9)	3.5 (2.75-4.50)	0.202
Recipient blood group, N (%)			
A	16 (20.3)	4 (28.6)	0.784
B	19 (24.1)	3 (21.4)	
O	44 (55.7)	7 (50)	
Anti-A/B titer, median (IQR)	16 (8-64)	32 (14-112)	0.228
HLA mismatch (mean ± SD)	3.44 ± 1.45	4.07 ± 1.14	0.129
rATG induction, N (%)	62 (78.5)	12 (85.7)	0.536
Antibody removal (plasmapheresis or IA or both)	66 (83.5)	13 (92.9)	0.369
PTDM, N (%)	27 (34.2)	06 (42.9)	0.532
Bacterial UTI, N (%)	31 (39.2)	04 (28.6)	0.448
Bacterial LRTI, N (%)	7 (8.9)	4 (28.6)	0.035
Other bacterial infections, N (%)	16 (20.3)	04 (28.6)	0.485
Fungal infections, N (%)	2 (2.5)	1 (7.1)	0.368
CMV disease, N (%)	6 (7.6)	1 (7.1)	0.953
BKV nephropathy, N (%)	7 (8.9)	0 (6.8)	0.247
Parvovirus infection, N (%)	2 (2.5)	1 (7.1)	0.368
Leukocytopenia, N (%)	15 (19.0)	3 (21.4)	0.831
DGF, N (%)	1 (1.3)	5 (35.7)	0.005
Graft loss, N (%)	2 (2.5)	6 (42.9)	0.005

**Table 7:** Comparison of outcomes between two cohorts of KTR as per initial anti-A/B titer BKV, BK virus; BPAR, biopsy-proven acute rejection; CMV, cytomegalovirus; DGF, delayed graft function; KTR, kidney transplant recipient; LRTI, lower respiratory tract infection; UTI, urinary tract infection.

Parameter	Titer ≤1:32 (N=64 (68.81%))	Titer >1:32 (N=29 (31.19%))	p-value
BPAR, N (%)	9 (14.1)	5 (17.2)	0.691
Bacterial UTI, N (%)	23 (35.9)	12 (41.4)	0.616
Bacterial LRTI, N (%)	7 (10.9)	4 (13.8)	0.693
Other bacterial infections, N (%)	16 (25.0)	04 (13.8)	0.223
Fungal infections, N (%)	3 (4.7)	0 (0)	0.236
CMV disease, N (%)	4 (6.3)	3 (10.3)	0.488
BKV nephropathy, N (%)	6 (9.4)	1 (3.4)	0.316
DGF, N (%)	1 (1.6)	5 (17.2)	0.004
Graft loss, N (%)	5 (7.8)	3 (10.3)	0.687

In the regression analysis, none of the factors, including recipient age, dialysis vintage, or the type of induction agent, predicted the incidence of BPAR. However, DGF was the only variable significantly associated with BPAR (OR: 43.33, 95% CI: 4.54-413.27, p<0.05). Human leukocyte antigen (HLA) mismatch showed a trend toward association (OR: 1.393, 95% CI: 0.90-2.14, p=0.132) but did not reach statistical significance (Table 8). Variables with p<0.2 on univariable analysis, namely DGF and HLA mismatch, were considered for a multivariable regression model. Given the limited number of BPAR (n=14), the model was restricted to two predictors to maintain an acceptable events-per-variable ratio and avoid overfitting.

**Table 8:** Univariable logistic regression analysis for predictor of BPAR BPAR, biopsy-proven acute rejection; DGF, delayed graft function; HLA, human leukocyte antigen; rATG, rabbit antithymocyte globulin.

Parameter	OR	95% CI	p-value
Recipient age	1.001	0.95-1.05	0.954
Re-transplantation	1.138	0.12-10.55	0.909
Dialysis vintage	1.035	0.97-1.10	0.284
Donor age	1.033	0.97-1.09	0.282
Anti-A/B titer >1:32	1.273	0.38-4.20	0.692
HLA mismatch	1.393	0.90-2.14	0.132
Extracorporeal antibody removal	2.561	0.30-21.31	0.385
rATG use	1.645	0.33-8.06	0.539

In multivariable logistic regression analysis, DGF remained an independent and significant predictor of BPAR, with an OR of 44.73 (95% CI: 4.52-441.74, p=0.001). HLA mismatch, while showing a trend toward association with rejection, did not reach statistical significance on adjusted analysis (OR: 1.455, 95% CI: 0.87-2.41, p=0.147). These findings suggest that DGF is a strong independent predictor of BPAR in ABOiKT. The wide CIs observed, particularly for DGF, reflect the limited number of rejection episodes in this cohort and should be interpreted with caution (Table 9).

**Table 9:** Multivariable logistic regression analysis for the predictor of BPAR BPAR, biopsy-proven acute rejection; DGF, delayed graft function; HLA, human leukocyte antigen.

Parameter	OR	95% CI	p-value
HLA mismatch	1.455	0.87-2.41	0.147
DGF	44.73	4.52-441.74	0.001

## Discussion

The art and science of ABOiKT have come a long way in the last two decades. In India, the number of ABOiKTs has gained momentum in the last 10 years. Our study is among the few studies published on ABOiKT in the Indian subcontinent with robust three-year follow-up data.

The mean age of the recipients in our study was 40.90 ± 11.94 years, which is similar to some earlier Indian studies [6,7]. As expected, 79 (84.9%) KTRs were males. The majority of the KTR (51, 54.8%) had blood group O. In our study, we did not find any significant correlation between any specific blood type and the incidence of BPAR or graft loss. In the study by Toki et al. [8], high anti-A/B antibody titers were predominant in blood group O (p<0.01), and the cumulative incidence of acute AMR was higher in the cohort with blood group O. It was hypothesized that O individuals might produce IgG against non-self antigens more readily than non-O individuals, accounting for a greater number of AMRs [9,10]. However, in a large cohort of 738 ABOiKTs, Montgomery et al. [11] did not find any significant difference in survival with respect to recipient and donor blood type. In the earlier phase of our study, rituximab was used at a higher dose of 500 mg as per the available consensus at that point in time. However, in subsequent studies, the low-dose rituximab regimen was found to be equally efficacious with a lower incidence of serious infections [12,13]. With the evolution of the art of ABOiKT, we did adapt and decreased the dose of rituximab to 200 mg. The median baseline anti-A/B antibody titer in our study was 32 (IQR: 8.64), and this was not found to be predicting BPAR. There is conflicting evidence about whether baseline anti-A/B antibody titer affects graft outcome or not. In many studies, baseline anti-A/B antibody titers were found to be poorer predictors of acute rejections [14,15]. In contrast, other studies found that ABOiKTR with high baseline titers experienced more acute rejection episodes and poor graft function in the follow-up period [16,17]. There are chances of rebound of titers after desensitization in cases with high baseline anti-A/B antibody titers [17]. The absence of an association between baseline anti-A/B antibody titer and acute rejection in our study may reflect the effectiveness of current desensitization protocols that could have minimized the impact of baseline antibody burden. The relatively small number of rejection events in our cohort may also have limited the statistical power to detect a modest association.

Infection is one of the most feared complications after ABOiKT. In our cohort, bacterial UTI (34, 37.6%) was more prevalent. It was comparatively higher than our earlier study in ABOcKT with rATG induction, where the incidence of bacterial UTI was around 27% [18]. The incidence of UTI in ABOiKT varies from 25% to 46% in some earlier Indian studies [6,7,19]. In the meta-analysis by de Weerd et al., infections as a cause of death were more common in ABOiKTR than in ABOcKTR (49% vs. 13%) [20]. The higher incidence of infection is thought to be due to the net cumulative immunosuppression in ABOiKTR. Factors such as rituximab use, plasmapheresis, and induction therapy over and above the standard maintenance immunosuppression do contribute to a somewhat higher incidence of infection-related complications. However, in some earlier comparative studies from India, there was no significant difference in the incidence of infections in ABOiKTR and ABOcKTR [21,22].

ABOiKT carries a higher risk of rejection due to pre-formed anti-ABO antibodies. However, with modern desensitization techniques and enhanced immunosuppression, successful outcomes are achievable. Hyperacute rejections involving anti-A/B blood group antibodies are rare nowadays. In our cohort, 14 (15.1%) KTRs developed BPAR episodes. Ten were AMRs, three were mixed, and one was ACR. We lost one graft by post-operative day 7, probably due to anti-A/B blood group antibody-mediated acute rejection due to rebound titers despite desensitization. Eight (8.6%) KTRs lost their graft. In the study by Jha et al. [6], the incidence of BPAR was 17%, of which 14% were ACR, and 3% were AMR. Similarly, in the study by Pawer et al. [7], the incidence of BPAR was 16%. Among them, 14.5% were AMR and 1.5% were ACR. In our previous cohort of ABOcKTR with rATG induction, the BPAR was 3.84% [18]. In another comparative study by Prabhakar et al. [19], the rate of BPAR in ABOiKTR and ABOcKTR was 28% and 18%, respectively. However, AMR was observed in 15% of the ABOiKTR cohort compared with 4% in the ABOcKTR cohort and was significantly higher (p<0.01). ACR was observed in 10% and 12% of recipients (p=0.4), respectively, and was comparable between groups. Several previous studies have shown that the rate of AMR is significantly higher in ABOiKT [20,23].

ABOiKT has historically been associated with higher risks of graft rejection and reduced survival rates compared to ABOcKT. However, advancements in desensitization protocols and immunosuppressive therapies have significantly improved outcomes for ABOiKT recipients. In our study, the rejection-free survival at six months, one year, and three years was 84 (90.3%), 83 (89.2%), and 79 (84.9%), respectively. Similarly, the overall graft survival at six months, one year, and three years was 90 (96.8%), 87 (93.5%), and 85 (91.45%), respectively. In an Indian study by Prabhakar et al. [19], the one-year and five-year graft survival rates after transplantation were 85% and 60% vs. 93.1% and 83% in ABOiKT and ABOcKT groups, respectively (p=0.03). The number of AMR was higher in the ABOiKT group, which could have contributed to the lower graft survival in their cohort. In the meta-analysis by de Weerd et al. [20], one-year uncensored graft survival of patients who were ABO-incompatible was 96% vs. 98% in ABO-compatible controls (RR: 0.97, 95% CI: 0.96-0.98, p=0.001). AMR (3.86, 95% CI: 2.05-7.29, p=0.001) was more common after ABOiKT. The three-year graft survival rate for ABO-incompatible recipients was 92%, compared with 94% for ABO-compatible recipients (p=0.04). Notably, grafts that survived beyond the first year showed comparable outcomes between ABO-incompatible and ABO-compatible groups, with a 97% survival rate between one- and three-year post-transplantation for both groups (p=0.70). A propensity-matched analysis comparing 296 ABOiKTRs with 1,184 ABO-compatible living donor recipients found that the rate of graft failure was higher in the ABO-incompatible group, with an HR of 2.63 (95% CI: 1.72-4.01) [24]. This underscores the need for careful patient selection and tailored immunosuppressive strategies in ABO-incompatible transplantation.

Both plasmapheresis and IA are effective desensitization strategies in ABOiKT, with comparable short-term outcomes [20,25]. IA offers advantages such as selectivity, preservation of plasma proteins, and potential cost savings through column reuse. However, the choice between these methods should be individualized, considering factors such as titer, patient condition, resource availability, and institutional protocols.

The ideal induction agent in ABOiKT is still not clear. Two commonly used induction agents are thymoglobulin and basiliximab. Direct comparative studies focusing exclusively on thymoglobulin vs. basiliximab induction in ABOiKT are limited. Pathak et al. [26] observed better patient survival rates with basiliximab than with thymoglobulin induction. The graft survival rates at one, three, and five years with basiliximab induction were 95.08%, 86.63%, and 85.24%, respectively, and with thymoglobulin induction were 80.65%, 70.97%, and 58.06%, respectively. In our study, we could not find any difference between nil/basiliximab and thymoglobulin induction as far as BPAR or graft loss is concerned. However, our study was not powered enough to conclude regarding the choice of induction agent in ABOiKT. Randomized controlled trials with greater sample sizes are needed to see whether basiliximab or thymoglobulin is the ideal induction agent in ABOiKT.

The incidence of DGF ranges from 4% to 10% in living donor transplants and from 5% to 50% in deceased donor kidney transplants [27]. In our cohort, it was 6.5%. Though the association between DGF and increased risk of BPAR is not well established, there are chances that DGF might increase the risk of rejection because of the persistent immune activation by ischemia-reperfusion injury. It can lead onto an upregulation of pro-inflammatory cytokines and increased expression of major histocompatibility complex antigens on tubular epithelial cells, thereby amplifying the adaptive immune response and lowering the threshold for alloimmune activation. In the context of ABO-incompatible transplantation, where the immune system is already sensitized and incompatible antibodies are present, DGF-induced inflammation might further amplify antibody-mediated injury, compounding the risk of rejection. Our study identified DGF as a strong independent predictor of graft loss following ABOiKT. Patients with DGF had approximately 43-fold higher adjusted odds of graft loss than those without DGF (adjusted OR: 43.33, 95% CI: 4.54-413.27, p<0.05). Although CI was wide, reflecting the relatively small number of BPAR or graft loss events and the consequent imprecision in the estimate, the association remained statistically significant, underscoring the adverse impact of DGF on graft survival. So, careful monitoring, vigilance, and a low threshold for kidney biopsy are needed in patients with DGF, especially in ABOiKT.

The present study is one of the few studies on ABOiKT published from the Indian subcontinent. We have data from a robust three-year follow-up, unlike the other studies. We tried to explore the caveat of the ideal induction agent in ABOiKT, i.e., a comparison between basiliximab and thymoglobulin.

However, the study has a few limitations. First, the retrospective nature of the study with a smaller sample size restricts the generalizability of the study. Second, we did not include a comparative analysis between ABOiKT and ABOcKT, which could have provided additional insight into graft outcomes and the incidence of infectious complications. Third, long-term graft survival, chronic AMR, and late infectious or cardiovascular outcomes might not have been adequately assessed due to relatively shorter duration of follow-up. Fourth, as it was a single-center study, outcomes may reflect institutional expertise and underrepresent adverse events observed in less experienced centers across the nation. Fifth, non-uniformity of rituximab dose and induction agent used could have affected the final interpretation of the results. Again, residual confounding cannot be excluded because this was an observational study. Although clinically relevant variables were considered, unmeasured factors such as donor-specific antibodies, HLA immunologic risk, post-transplant anti-A/B antibody rebound, variability in immunosuppressive drug exposure, and differences in the intensity of desensitization may have influenced the outcomes.

## Conclusions

ABOiKT has emerged as a viable solution to address the growing organ shortage, allowing for successful transplants across blood group barriers. Despite the inherent risk of AMR due to preformed anti-ABO antibodies, the advancements in desensitization protocols and targeted immunosuppressive therapies have significantly improved graft survival rates. In our cohort, DGF was found to be significantly associated with rejection episodes, highlighting the importance of strategies aimed at preventing and optimizing the management of DGF. Future studies should focus on refining the immunosuppressive protocols, improving cost-effectiveness, and reducing the burden of long-term complications. With continued advancements, ABOiKT has the potential to further expand the donor pool with acceptable long-term patient and graft survival.
